# Morphology of nasal-cavity tumours in rats after chronic inhalation of 1,2-dibromo-3-chloropropane.

**DOI:** 10.1038/bjc.1980.311

**Published:** 1980-11

**Authors:** G. Reznik, H. Reznik-Schüller, J. M. Ward, S. F. Stinson

## Abstract

**Images:**


*Br. J. Cancer* (1980) **42**, 772

### MORPHOLOGY OF NASAL-CAVITY TUMOURS IN RATS AFTER CHRONIC INHALATION OF 1,2-DIBROMO-3-CHLOROPROPANE

G. REZNIK*, H. REZNIK-SCHÜLLER†, J. M. WARD* AND S. F. STINSON*

*From the *Tumor Pathology Branch, Carcinogenesis Testing Program, National Cancer Institute National Toxicology Program, Bethesda, Md* 20205, *and †Chemical Carcinogenesis Program, N.C.I.-Frederick Cancer Research Center, Frederick, Md* 21701, *U.S.A.*

Received 21 March 1980 Accepted 18 July 1980

**Summary.—Groups of 50 F344 rats of each sex were exposed to 0·6 or 3·0 pts/$10^6$ of 1,2-dibromo-3-chloropropane (DBCP) by inhalation for 6 h/day, 5 days/week for 103 weeks. Fifty rats of each sex inhaling filtered air were used as unexposed controls. All survivors were killed at 104 weeks. Up to 93% of the male and female rats developed neoplasms of the nasal cavity. Most of the tumours were adenomas, squamous-cell papillomas, squamous-cell carcinomas, and adenocarcinomas. In the low-dose group 78% of the tumours in males and 66% in females were benign, whereas in the high-dose groups 89% in males and 76% in females were malignant. Invasion through the cribriform plate into the cerebrum or metastasis to the regional lymph nodes was found in 73% of the carcinomas in males and 51% in females. Electron-microscopic examination suggested that the basal cells of the olfactory epithelium were the site of origin of the poorly differentiated adenocarcinomas.**

THE SOIL FUMIGANT NEMATOCIDE 1,2-dibromo-3-chloropropane (DBCP) has been manufactured in the United States for over 15 years and is used primarily for soybeans, grapes, citrus, pineapples, and peaches, in amounts totalling about 12–15 million pounds annually (NIOSH, 1977; CFR, 1977). Torkelson *et al.* (1961) found the compound to be moderately to highly toxic in rats on repeated exposure, producing damage to the lung, liver and testes at concentrations as low as 5 pts/$10^6$. DBCP was found to be mutagenic to *Salmonella typhimurium* (TA 1530) and *Escherichia coli* (Pol. A) by Rosenkranz (1975). Prival *et al.* (1977) reported DBCP to be a direct weak mutagen in *Salmonella typhimurium* (TA 1535) and Blum & Ames (1977) and others (Biles *et al.*, 1979; Stolzenberg & Hine, 1979) presented data that DBCP was mutagenic with metabolic activation in Salmonella TA 100. Biles *et al.* (1979) showed the influence of contaminants on the mutagenic activity of DBCP. Teramoto *et al.* (1980) induced dominant lethals in rats in the post-meiotic stage of spermatogenesis, especially in the early spermatid stage. However, DBCP did not cause dominant lethals in mice.

DBCP was carcinogenic in rats and mice when the substance was administered by chronic oral intubation (Olson *et al.*, 1973). When applied to mouse skin, DBCP led to a highly significant incidence of tumours of the lung and stomach (Van Duuren *et al.*, 1979). DBCP was not teratogenic, but a foetotoxic effect was observed in Wistar rats (Ruddick & Newsome, 1979). In the same study, it was shown that fat, when compared with all other tissues, always contained the highest amount of DBCP.

Recently DBCP has been implicated as the possible cause of sterility in individuals with histories of industrial exposure to this chemical (Whorton *et al.*, 1979; Marshall *et al.*, 1978; Biava *et al.*, 1978; Potashnik *et al.*, 1978). The major effects were azoospermia or oligospermia. Sandifer *et al.* (1979) showed significant differences in median sperm counts among formulators, custom applicators, and farmers.

Because of some of these observations in animals and in man, DBCP was selected for testing by the Carcinogenesis Testing Program of the National Cancer Institute. Since inhalation is the most common route of exposure to humans, this route was chosen for the rodent bioassay.

### MATERIALS AND METHODS

Technical grade DBCP was obtained from the Shell Chemical Co., San Ramon, California, and had a purity of 96%. It contained allyl chloride (0·7%) and epichlorohydrin (0·8%) and a residue of 2·5% as impurities. Liquid DBCP was generated as a vapour by bubbling metered, filtered, dried air, regulated at 10 psi, through a 1l glass globe flask, wrapped with black tape to reduce light exposure and containing at least 500 ml of the test chemical. The resultant vapour was forced into the inhalation chambers. Each chamber had a separate flask and generation system. Each flask was suspended in a Plexiglass box and equipped with an air line attached to its chamber exhaust duct and was under negative pressure with respect to the chamber room. The inhalation chambers were continuously monitored, and concentrations were determined 4 times per day. The chamber concentrations were usually within 10% of the desired concentration. The mean concentrations were $0{\cdot}59 \pm 0{\cdot}08$ pts/$10^6$ (low dose) and $2{\cdot}87 \pm 0{\cdot}42$ pts/$10^6$ (high dose).

Four-week-old F344 rats were obtained from the NCI–Frederick Cancer Research Center (Frederick, Md). All animals were observed for 1 week before the start of the experiment and randomized into groups. Male rats were housed 3 per cage and female rats 4 per cage. The cages were suspended on aluminium racks inside the inhalation chambers. The food was placed in the chambers 1 h after the end of the DBCP exposure period each weekday, and was removed the following morning before the start of the exposure period. Food was available *ad libitum* on weekends. Water was available from water bottles equipped with stainless-steel tubes. The animals lived in the inhalation chambers continuously (whole-body exposure) except when being weighed. Control groups lived in inhalation chambers in the same room, and were exposed to filtered, conditioned air. Air flow into the glass and stainless steel inhalation chambers was maintained at 1000 l/min.

A subchronic inhalation study was done using F344 rats to determine the concentrations of DBCP vapour to be used in the chronic study. Groups of 5 male and female rats were exposed to 1, 5 or 25 pts/$10^6$ for 6 h/day, 5 days/week for 13 weeks. As a result of this study, doses for the chronic experiments were set at 0·6 and 3·0 pts/$10^6$.

The test groups, doses administered, and duration of the chronic studies are shown in the Table. Moribund animals, and those that survived to the termination of the study were killed and necropsied. Gross and microscopic examinations were performed on all major tissues. Tissues were fixed in 10% neutral buffered formalin, embedded in paraffin, sectioned (6 $\mu$m) and routinely stained with haematoxylin and eosin. Heads were fixed whole in the formalin, and later in Bouin's solution, and decalcified by Perenyi's method (Emmel & Cowdry, 1964). Step cross-sections, in a dorso–ventral plane perpendicular to the long axis of the skull, were taken from the nostrils to the olfactory lobes of the brain to ensure adequate tissue sampling and to enable visualization of the extent of neoplastic growth into the brain. An occasional section was subjected to special staining techniques (PAS, toluidine blue and van Gieson) for more definitive diagnosis.

For electron microscopic examination 5 animals at the 0·6 pts/$10^6$ dose level were anaesthetized by i.p. injection of sodium pentobarbital (Diabutal, Diamond Laboratories, Des Moines, IA). They were then perfused *in situ via* the portal vein with 5% dextrose solution followed by a fixative solution of 2% cacodylate-buffered glutaraldehyde. Tissue samples from one macroscopically visible tumour from the ethmoturbinal region which had invaded the brain and the subcutis of the nose were excised and immersed for an additional 2 h in the fixative. They were then washed in cacodylate buffer, post-fixed for 2 h in 1% cacodylate-buffered $OsO_4$, dehydrated in ascending concentrations of ethanol, and embedded in Epon 812 (Ladd Research Industries, Burlington, VT). Sections were cut on an LKB ultrotome III (LKB, Bromma, Sweden), mounted on uncoated copper grids, stained with uranyl acetate and lead citrate, and examined in a

Phillips 201 C electron microscope at an accelerating voltage of 60 kV.

Data on this experiment were recorded in the Carcinogenesis Bioassay Data System (Linhart *et al.*, 1974). The one-tailed Fisher exact test was used to compare the tumour incidence of control groups and those exposed to DBCP (Cox, 1970).

### RESULTS

In the subchronic study, 2 female rats receiving the 25-pts/$10^6$ dose died and 3 (2 females, 1 male) rats were moribund when killed. Histopathological findings associated with subchronic inhalation of 25 pts/$10^6$ of DBCP were focal atrophy of the olfactory epithelium and megalocytosis of the basal cells of the respiratory epithelium of the nasal cavity, meningo-encephalitis, increased vacuolization or necrosis of the cortical epithelium of the adrenal gland, focal necrosis of the liver accompanied by hepatic regeneration, degenerative and regenerative changes of the proximal and distal tubular epithelia of the kidney accompanied by megalocytosis, necrosis of the tracheal and bronchial epithelium and squamous metaplasia of the bronchial epithelium, and atrophy of the testes with hypo-spermatogenesis and multinucleated giant spermatids. Male and female rats receiving the 1- or 5-pts/$10^6$ doses had megalocytic epithelia in the proximal tubules of the kidney.

In the chronic inhalation study, the mortality of rats indicate significant differences ($P < 0{\cdot}001$) in both sexes, due to shorter survival of the high dose group in each sex than of the low-dose and control groups. In male rats (19/50) 38% of the control group, (42/50) 84% of the low dose, and (5/49) 10% of the high-dose group lived to the end of the study. In females (20/50) 40% of the control, (40/50) 80% of the low-dose and (6/51) 12% of the high-dose group lived to the end of the study.

Neoplasms related to inhalation of DBCP were seen in the low- and high-dose groups in male and female rats, in the nasal cavity, tongue, pharynx, larynx and kidney. Because the nasal cavity was the main target organ, this paper describes the morphology of these lesions in detail. The spectrum of nasal-cavity neoplasms consisted of adenomas, squamous-cell papillomas, squamous-cell carcinomas and adenocarcinomas. The number of rats with tumours in the nasal cavity is given in the Table. The percentage of animals with neoplasms in the nasal cavity was very high in both sexes of both the low- and high-dose groups. The total number of tumours showed a dose-dependency. The incidence of benign neoplasms in each group decreased with increasing dose, while the incidence of carcinomas increased in both male and female rats. While in the low-dose group only 3 rats (1 male and 2 females) showed brain infiltration by tumour tissue, 33/40 high-dose male rats and 22/37 female rats with nasal-cavity carcinomas demonstrated invasion of the brain, or metastasis to regional lymph nodes (3/37). The benign neoplasms (adenomas, and squamous-cell papillomas) and some of the squamous-cell

TABLE.—*Experimental design and results of F*344 *rats exposed to* 1,2-*dibromo*-3-*chloropropane vapour* 6 *h/day*, 5 *days/week for* 103 *weeks*

| DBCP concentration (pts/$10^6$) | Sex | Effective no. of animals | No. of animals (and %) with nasal-cavity neoplasms | | | |
|---|---|---|---|---|---|---|
| | | | Total | Benign | Malignant | Invasive or metastasizing |
| 0 | M | 50 | 0 | 0 | 0 | 0 |
| | F | 50 | 1 (2) | 1 (2) | 1 (2) | 0 |
| 0·6 (low dose) | M | 50 | 42 (84) | 39 (78) | 7 (14) | 1 (2) |
| | F | 50 | 35 (70) | 33 (66) | 3 (6) | 2 (4) |
| 3·0 (high dose) | M | 45 | 42 (93) | 15 (33) | 40 (89) | 33 (73) |
| | F | 49 | 45 (92) | 25 (51) | 37 (76) | 25 (51) |

FIG. 1.—Papillary adenomas (arrowed) in the nasal cavity of a female F344 rat that inhaled DBCP (low dose). S = septum; NT = nasoturbinal, MT = Maxilloturbinal, ND = naso-lacrimal duct, I = incisor. H. & E. ×32.

FIG. 2.—Higher magnification of Fig. 1, showing the glandular pattern with uniform cell nuclei and partially vacuolated cytoplasm. H. & E. ×265.

carcinomas were located in the anterior part of the nasal cavity, in the region of the respiratory turbinals (naso- and maxilloturbinals) whereas the adenocarcinomas and most of the squamous-cell carcinomas were located in the region of the ethmoturbinals and the posterior part of the nasal septum. Adenomas varied in size, and originated from the epithelium of the naso- and maxilloturbinals or the nasal septum, and projected into the lumen of the nasal cavity (Fig. 1). Occasionally it was difficult to find the cell of origin because the submucosal nasal glands were involved in the tumour growth. Usually the tumours were small, and grew along the structures of the nasal cavity without invasion (Fig. 2). The adenomas were composed of well differentiated cells forming glands with PAS-positive secretions in the lumen. Areas of squamous differentiation were occasionally seen. Most of the animals had multiple adenomas, and often the rats showed adenocarcinomas and adenomas at the same time, but in different regions of the nasal cavity.

The adenocarcinomas were exophytic and endophytic, showing cellular pleomorphism and atypia. In the most advanced stages, the neoplasms filled one or both sides of the nasal cavities, destroying them by invasion into the surrounding bones, the vessels, cribriform plate and the brain (Figs 3–6). The cells were arranged in different patterns from well to poorly differentiated, containing bizarre mitotic figures. The neoplasms demonstrated dark and light cells with hyperchromatic round to oval nuclei. When these tumours invaded the brain, they often showed pseudo-rosettes, rosettes or glandular formations (Fig. 7). In some instances, focal squamous metaplasia was present within these neoplasms. The origin of these

FIG. 3.—Invasive adenocarcinoma in the region of the ethmo-turbinals of a male rat (low dose) (NM = nasopharyngeal meatus). H. & E. ×8.

FIG. 4.—Adenocarcinoma in a female rat (low dose) invading lymphatics in the submucosa of the nasopharyngeal meatus (NM). H. & E. ×32.

tumours could not be determined by the histomorphological methodology used.

Squamous-cell papillomas (14% in low-dose and 6% in high-dose males, and 20% in low-dose and 6% in high-dose females) were composed of acanthotic epithelium developed from respiratory epithelium, or from the normal squamous epithelium lining of the most anterior part of the nasal entrance and nasal cavity. The papillomas were often multiple, and varied in size from animal to animal.

Squamous-cell carcinomas (8% in the low-dose and 37% in high-dose males and 10% in both low-dose and high-dose females) were invasive (Fig. 8) forming fronds or finger-like structures and nests of anaplastic cells in underlying tissues (Fig. 9), masses of keratin and, often, invasion to the cerebrum (Fig. 10). These tumours also showed pseudo-rosettes. Although these tumours frequently invaded the brain and vessels, metastasis to cervical lymph nodes was only seen in 2 cases (high-dose females). A bizarre tumour composed of giant cells, often multinucleated and elongated, was found in one animal. It was the only one seen of its type, and was diagnosed as carcinosarcoma.

In addition to neoplastic changes, the nasal cavities of almost all exposed rats contained focal or multifocal hyperplastic areas and squamous metaplasia or dysplasia of the squamous or respiratory epithelium of the nasal cavity. Such changes were also diagnosed in the epithelium of the glandular ducts or acini of the submucosal glands of the nasal cavity.

The tumour that was examined by electron microscopy had infiltrated the brain and had grown through the nasal bone to the subcutis of the nose. It was diagnosed as a poorly differentiated

FIG. 5.—Adenocarcinoma in a male rat (low dose) destroying bones and invading the brain. H. & E. ×105.

FIG. 7.—High mitotic activity and rosette formation in an adenocarcinoma of a female rat (low dose) that invaded the cerebrum. H. & E. ×700.

FIG. 6.—Longitudinal section through the brain of a female rat (low dose) with invasion of an adenocarcinoma (arrows) into the olfactory lobe and cerebrum (C). H. & E. ×8·5.

FIG. 8.—Squamous-cell carcinoma in the respiratory region of the nasal cavity of a male rat (low dose) infiltrating the nasal and maxillary bone (left). On the right side an adenocarcinoma has destroyed the nasoturbinal. H. & E. × 9.

FIG. 9.—Higher magnification of the well-differentiated squamous-cell carcinoma (described in Fig. 8). H. & E. × 265.

adenocarcinoma by standard light microscopy, and was considered a typical example of this tumour type on the basis of its histological figures. Ultrastructurally, the oval to elongated tumour cells were often arranged around a narrow lumen, whilst adjacent cells were connected to one another by junctional complexes. This gland-like growth pattern was apparent in all parts of the neoplasm. All tumour cells had numerous polyribosomes in their cytoplasm, whilst rough endoplasmic reticulum was only sparse. The mitochondria were moderately swollen and demonstrated partial loss of their cristae. Those tumour cells which had infiltrated through the nasal bone to the subcutis of the nose demonstrated the highest grade of cytoplasmic differentiation, with long slender microvilli lining their lumina. At all other sites of the neoplasm these organelles were lacking. The oval nuclei of tumour cells in the subcutis of the nose were rich in heterochromatin, whilst in the ethmoturbinal region and brain their nuclei were markedly lobulated, and demonstrated little heterochromatin, most of which was condensed marginally. Moreover, the nuclear–cytoplasmic ratio was increased in the latter 2 areas. In the region of the ethmoturbinals many tumour cells had very large nucleoli, a feature not noticed in other areas. Ultrastructural characteristics indicating a neurogenic origin (*e.g.* neurosecretion axons, neurotubules) were not found in any of the tumour cells. Ciliogenesis and features of mucous secretion, which would have suggested tumour origin from the nasal respiratory epithelium, were not found.

### DISCUSSION

In a previous study, DBCP administered by gavage was found to be carcinogenic for Osborne–Mendel rats and

FIG. 10.—Squamous-cell carcinoma of a female rat (low dose) infiltrating the olfactory lobe and cerebrum. H. & E. ×32.

B6C3F1 mice, inducing squamous-cell carcinomas of the forestomach in animals of both sexes (Olson *et al.*, 1973; Technical Report Series No. 28, 1978). Our study confirms the carcinogenicity of DBCP in rats, but also demonstrates that, with the inhalation route of exposure, the main target organ for this compound is the nasal cavity. Some recent papers have shown that DBCP is readily absorbed from the rat gastrointestinal tract when given by gavage (Kato *et al.*, 1979*a*). It has also been demonstrated that radiocarbon from $^{14}$C-DBCP was incorporated into proteins after activation by the microsomal oxidase system (Kato *et al.*, 1979*b*). Although it was reported by several authors that excessive exposure of animals to DBCP by various routes caused injury to the liver, kidneys and testes, lesions in man have been found localized to the testes (Whorton *et al.*, 1979; Marshall *et al.*, 1978). Because man is usually exposed to the substance by inhalation, lesions should be expected in the respiratory tract. Most of the reports in man, however, as well as the animal studies described in the literature, did not mention any lesions in the nasal cavities. When applied to mouse skin, DBCP produced a highly significant incidence of tumours of the lung and stomach, but it was not carcinogenic at the site of application (Van Duuren *et al.*, 1979). This indicates that DBCP, though acting locally in the rat and mouse forestomach and nasal cavity, may be metabolized differently in the mouse skin.

In our inhalation experiment with DBCP, rats of the high-dose groups showed a high percentage of malignant nasal-cavity tumours. Most of these carcinomas penetrated the cribriform plate and invaded the cerebrum. Occupational exposure to certain chemicals leads to the appearance of carcinomas in the nasal cavity of man (Acheson *et al.*, 1968; Doll, 1958; Hadfield, 1970). Some other environmental carcinogens fed to rats have also induced nasal-cavity tumours. P-Cresidine, used in the production of various azo dyes, induced dose-related olfactory neuroblastomas in male and female rats (Technical Report Series, No. 142, 1979). 1,4-Dioxane, used extensively as an industrial solvent for lacquers, varnishes, paints, plastics, dyes, oils, waxes and resins, induced squamous-cell carcinomas and adenocarcinomas in up to 47% of male and female rats (Technical Report Series, No. 80, 1978). Low incidences of neuroepitheliomas and carcinomas in the nasal cavities were induced by thio-TEPA, an ethyleneimine alkylating agent that was introduced for clinical use in cancer chemotherapy (Technical Report Series, No. 58, 1978). Procarbazine, a methylhydrazine derivative which has been shown to have antineoplastic activity in advanced Hodgkin's disease and in oat-cell carcinoma of the lung, induced carcinomas and neuroblastomas of the nasal cavity of rats (Technical Report Series, No. 19, 1979). More recent studies with 1,2-dibromoethane, used as a fumigant and gasoline additive, induced a high percentage of nasal-cavity carcinomas in rats (Technical Report Series, No. 206, 1980). Other experiments revealed a high percentage of nasal-cavity carcinomas in rats exposed to various nitrosamines (Reznik *et al.*, 1975; Althoff *et al.*, 1974). The diagnoses of the neoplasms ranged from squamous-cell papillomas to neuroesthesioblastomas, and the problems in diagnosing these tumours were manifold. Poorly differentiated carcinomas with rosette and pseudo-rosette formation are often misdiagnosed as neuroesthesioblastomas because of the uncertain origin in the region of the ethmoturbinals. As in our study, these neoplasms penetrated the cribriform plate and invaded the brain, where they formed differentiated areas, squamous foci, or rosettes and pseudo-rosettes with a high cellularity and mitotic rate. All these studies demonstrate quite convincingly that the nasal cavity has a high sensitivity to a wide range of environmental carcinogens, and should hence be routinely examined, grossly and histologically, in all standard rodent carcinogenesis bioassays.

In theory, epithelial tumours arising in the nasal cavity could be derived from cells of the respiratory epithelium, the olfactory epithelium, or from cells of the submucosal glands. Ciliated and mucous cells from the respiratory epithelium, as well as cells from the submucosal glands, can be excluded as possible sites of tumour origin, as none of the tumour cells displayed characteristic features of these cells. Similarly, olfactory sensory cells and their precursors (neuroblasts (Schade, 1973)) are also highly unlikely as a source of the neoplasms, because the tumour cells essentially lacked any characteristics of neurogenic cells. Long slender microvilli in a nonsecreting cell, as found in the well differentiated parts of this nasal tumour, are typical features of the olfactory sustentacular cell (Yamamoto, 1976; Frisch, 1967). On the other hand, smooth endoplasmic reticulum, which represents another typical organelle of mature sustentacular cells, was not found in any of the neoplastic cells. In contrast, abundant polyribosomes and scanty rough endoplasmic reticulum were prominent in all tumours cells. These characteristics, and a high nuclear–cytoplasmic ratio, are typical of the basal cells of the olfactory epithelium (Yamamoto, 1976; Seifert & Ule, 1967). This cell type has been suggested as the stem cell from which olfactory sustentacular cells develop through differentiation (Yamamoto, 1976; Seifert & Ule, 1967). In view of this, the nasal-cavity tumour that was examined ultrastructurally appears to be an adenocarcinoma arising from the olfactory basal cells and displays various stages of their differentiation towards mature olfactory sustentacular cells.

We acknowledge the assistance of Robert Nye, Jack Romine, Jean Keller, Amelia Grant and Borge Ulland.

REFERENCES

ACHESON, E. D., COWDELL, R. H., HADFIELD, E. H. & MACBETH, R. G. (1968) Nasal cancer in wood workers in the furniture industry. *Br. Med. J.*, ii, 587.

ALTHOFF, J., HILFRICH, J., KRÜGER, F. W. & MOHR, U. (1974) The carcinogenic effect of 2-oxo-propyl-propylnitrosamine in Sprague-Dawley rats. *Z. Krebsforsch*, **81**, 23.

BIAVA, C., SMUCKLER, E. & WHORTON, D. (1978) The testicular morphology of individuals exposed to dibromochloropropane. *Exp. Mol. Pathol.*, **29**, 448.

BILES, R. W., CONNOR, T. H., TRIEFF, N. M. & LEGATOR, M. S. (1979) The influence of contaminants on the mutagenic activity of dibromochloropropane (DBCP). *J. Environ. Pathol. Toxicol.*, **2**, 301.

BLUM, A. & AMES, B. (1977) Flame retardant additives as possible cancer hazards. *Science*, **195**, 17.

TECHNICAL REPORT SERIES No. 28 (1978) Bioassay of dibromochloropropane for possible carcinogenicity. US DHEW, PHS, NIH, NCI. Washington, DC: U.S. Govt Print. Off. p. 86.

TECHNICAL REPORT SERIES No. 80 (1978) Bioassay of 1,4-dioxane for possible carcinogenicity. US DHEW, PHS, NIH, NCI. Washington, DC: U.S.Govt Print. Off. p. 108.

TECHNICAL REPORT SERIES No. 58 (1978) Bioassay of Thio-Tepa for possible carcinogenicity. US DHEW, PHS, NIH, NCI. Washington DC: U.S. Govt Print. Off. p. 168.

TECHNICAL REPORT SERIES No. 142 (1979) Bioassay of p-cresidine for possible carcinogenicity. US DHEW, PHS, NIH, NCI. Washington DC: U.S. Govt Print. Off. p. 63.

TECHNICAL REPORT SERIES No. 19 (1979) Bioassay of procarbazine for possible carcinogenicity.

US DHEW, PHS, NIH, NCI. Washington, DC: US Govt Print. Off. p. 124.

TECHNICAL REPORT SERIES No. 206 (1980) Bioassay of 1,2-dibromoethane for possible carcinogenicity. U.S. DHEW, PHS, NIH, NCI. Washington DC: U.S. Govt Print. Off. p. 100.

CODE OF FEDERAL REGULATIONS (CFR) (1977) **40**, 180.

COX, D. R. (1970) *Analysis of Binary Data*. London: Methuen. p. 48.

DOLL, R. (1958) Cancer of the lung and nose in nickel workers. *Br. J. Indust. Med.*, **15**, 217.

EMMEL, M. & COWDRY, E. V. (1964) *Laboratory Technique in Biology and Medicine*. 4th Ed. Baltimore: Williams & Wilkins. p. 333.

FRISCH, D. (1967) Ultrastructure of mouse olfactory mucosa. *J. Anat.*, **121**, 87.

HADFIELD, E. H. (1970) A study of adenocarcinoma of the paranasal sinuses in wood workers in the furniture industry. *Ann. R. Coll. Surg.*, **46**, 301.

KATO, Y., MATANO, O. & GOTO, S. (1979*a*) Covalent bindings of DBCP *in vitro*. *Toxicol. Lett.*, **3**, 299.

KATO, Y., SATO, K., MAKI, S., MATANO, O. & GOTO, S. (1979*b*) Metabolic fate of 1,2-dibromo-3-chloropropane (DBCP) in rats. *J. Pesticide Sci.*, **4**, 195.

LINHART, M. S., COOPER, J. A., MARTIN, R. L., PAGE, N. P. & PETERS, J. A. (1974) Carcinogenesis bioassay data system. *Comp. Biomed. Res.*, **7**, 230.

MARSHALL, S., WHORTON, D., KRAUSS, R. & PALMER, W. (1978) Effect of pesticides on testicular function. *Urology*, **11**, 257.

MORGAN, J. G. (1958) Some observations on the incidence of respiratory cancer in nickel workers. *Br. J. Indust. Med.*, **15**, 224.

NATIONAL INSTITUTE FOR OCCUPATIONAL AND ENVIRONMENTAL SAFETY AND HEALTH (1977) *A Recommended Standard for Occupational Exposure to dibromochloropropane*. U.S. Dept. of Health, Education and Welfare. Washington, D.C.: U.S. Govt Print. Off. p. 12.

OLSON, W. A., HABERMANN, R. T., WARD, J. & WEISBURGER, J. (1973) Induction of stomach cancer in rats and mice by halogenated aliphatic fumigants. *J. Natl Cancer Inst.*, **51**, 1993.

POTASHNIK, G., BEN-ADERET, N., ISRAELI, R., YANAI-Inbar, L. & SOBER, L. (1978) Suppressive effect of 1,2-dibromo-3-chloropropane on human spermatogenesis. *Fertil. Steril.*, **30**, 444.

PRIVAL, M., MCCOY, E. & ROSENKRANZ, H. (1977) Tris (2,3-dibromopropyl) phosphate: Mutagenicity of a widely used flame retardant. *Science*, **195**, 76.

REZNIK, G., MOHR, U. & KRÜGER, W. (1975) Carcinogenic effects of di-n-propyl-nitrosamine, beta-hydroxypropyl-n-propylnitrosamine and methyl-n-propylnitrosamine in Sprague–Dawley rats. *J. Natl Cancer Inst.*, **54**, 937.

ROSENKRANZ, H. (1975) Genetic activity of 1,2-dibromo-3-chloropropane, a widely-used fumigant. *Bull. Environ. Contam. Roxicol.*, **14**, 8.

RUDDICK, J. A. & NEWSOME, W. H. (1979) A teratogenicity and tissue distribution study on dibromochloropropane in the rat. *Bull. Environ. Contam. Toxicol.*, **21**, 438.

SANDIFER, S. H., WILKINS, R. T., LOADHOLT, C. B., LANE, L. G. & ELDRIDGE, J. C. (1979) Spermatogenesis in agricultural workers exposed to dibromochloropropane (DBCP). *Bull. Environ. Contam. Toxicol.*, **23**, 703.

SCHADE, A. R. (1973) Sinneszellen und Nervenzellen. In: *Grundlagen der Zytologie*. Eds Hirsch *et al.* Stuttgart: Gustav Fischer Verlag. p. 579.

SEIFERT, K. & ULE, G. (1967) Die Ultrastruktur der Reichschleimhaut der neugeborenen und jugendlichen weissen Maus. *Z. Zellforsch*, **76**, 147.

STOLZENBERG, S. J. & HINE, C. H. (1979) Mutagenicity of halogenated and oxygenated three-carbon compounds. *J. Toxicol. Environ. Health*, **5**, 1149.

TERAMOTO, S., SAITO, S., AOYAMA, H. & SHIRASU, Y. (1980) Dominant lethal mutation induced in male rats by 1,2-dibromo-3-chloropropane (DBCP) *Mutat. Res.*, **77**, 71.

TORKELSON, T., SADEK, S., ROWE, V. & 4 others (1961) Toxicologic investigations of 1,2-dibromochloropropane. *Toxicol. Appl. Pharmacol.*, **3**, 545.

VAN DUUREN, B. L., GOLDSCHMIDT, B. M., LOEWENGART, G. & 4 others (1979) Carcinogenicity of halogenated olefinic and aliphatic hydrocarbons in mice. *J. Natl Cancer Inst.*, **63**, 1433.

WHORTON, D., MILBY, T., KRAUSS, R. & STUBBS, H. A. (1979) Testicular functions in DBCP exposed pesticide workers. *J. Occup. Med.*, **21**, 161.

YAMAMOTO, A. (1976) An electron microscopic study of the olfactory epithelium in the bat and rabbit. *Arch. Histol. Jap.*, **38**, 359.


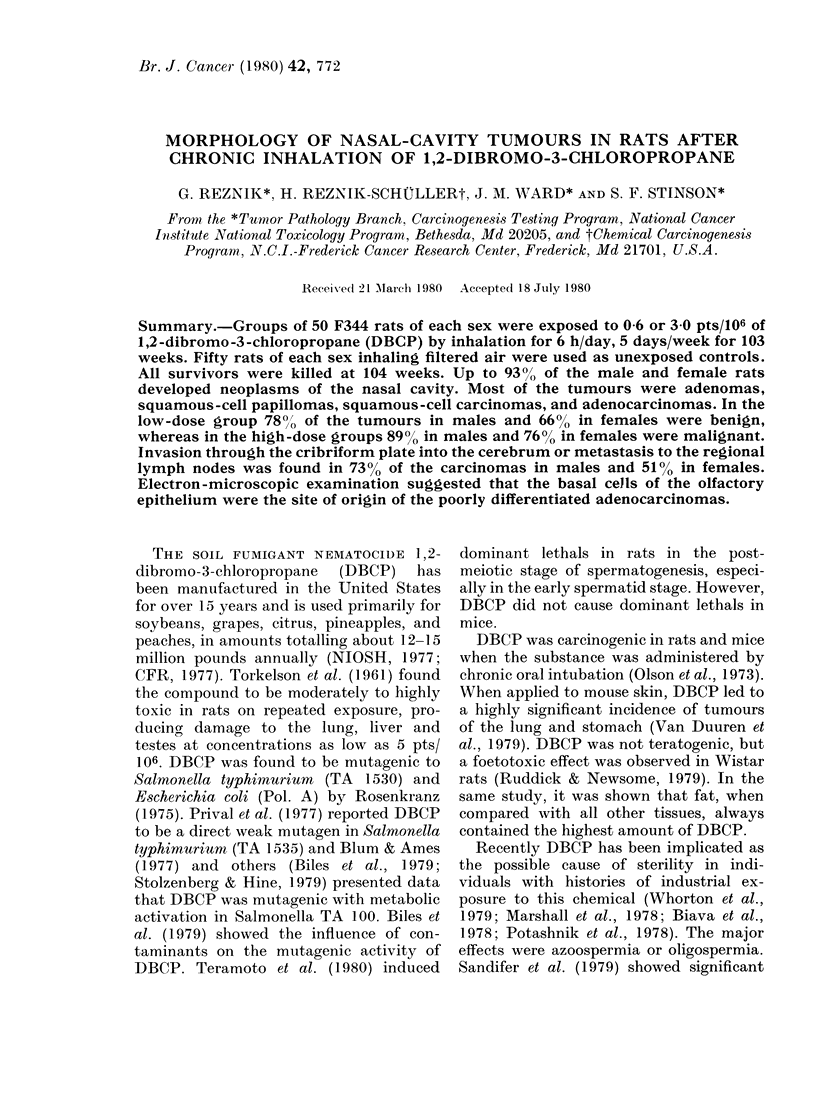

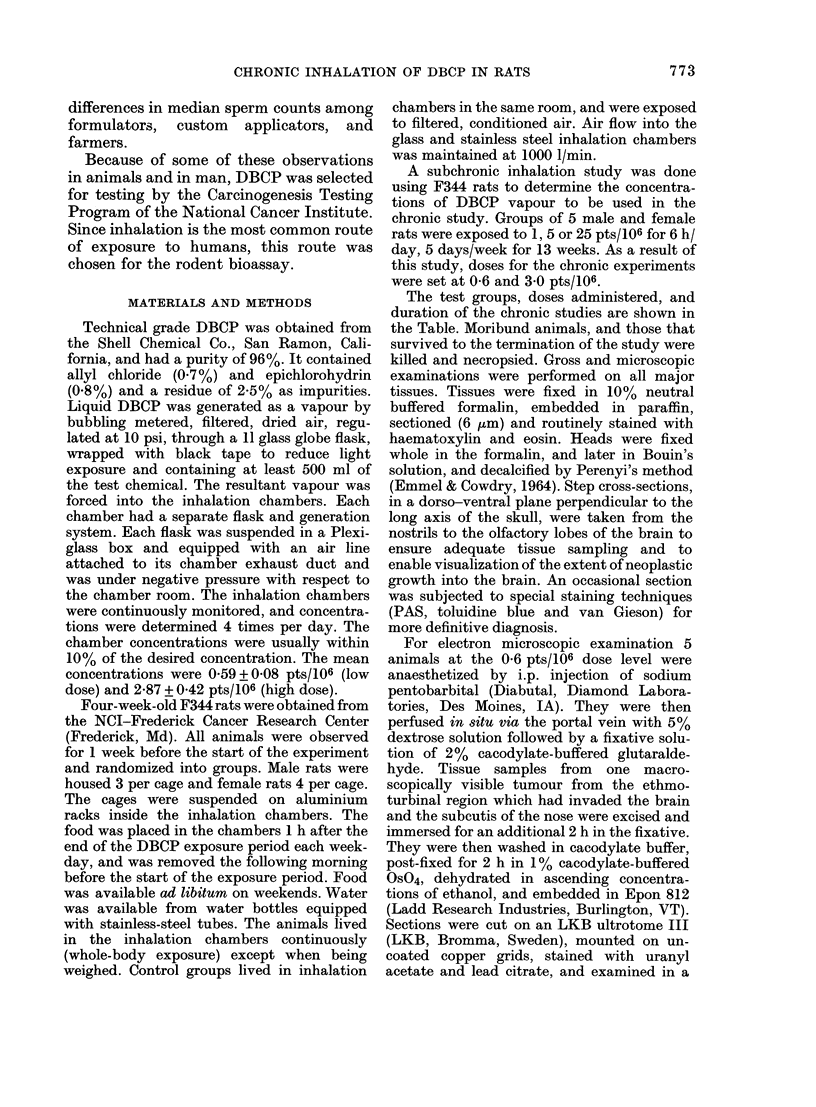

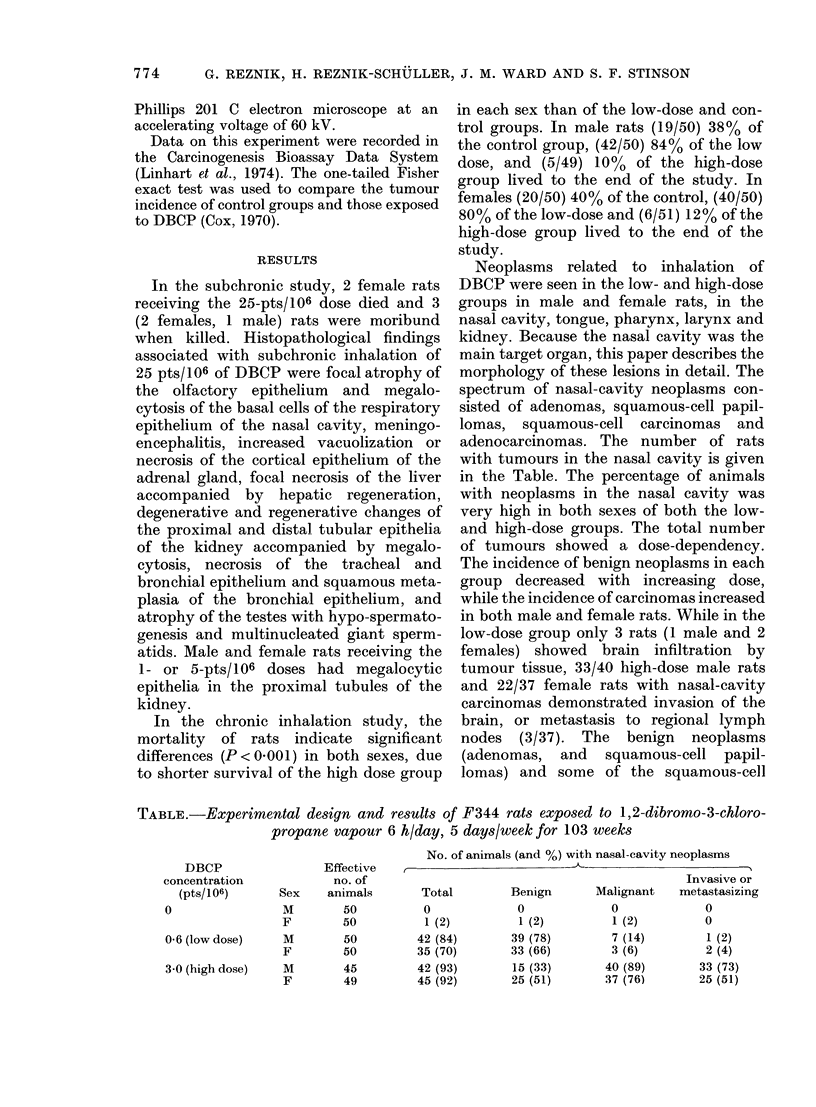

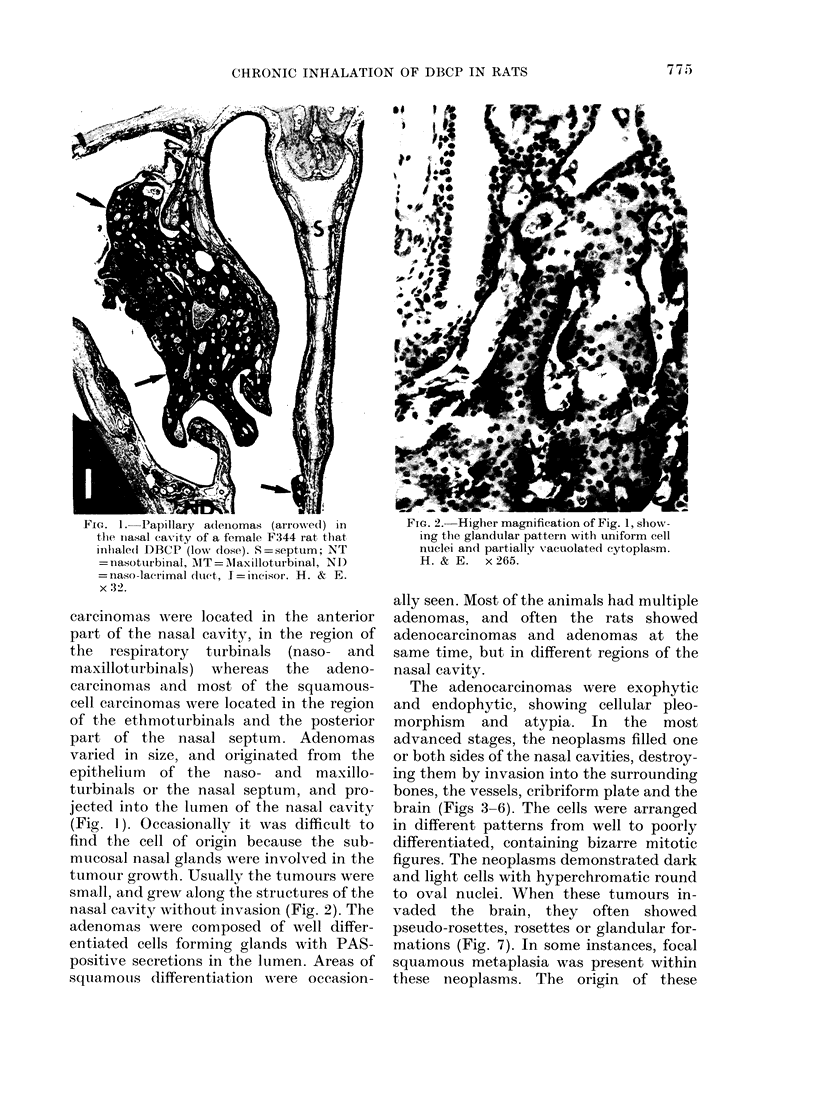

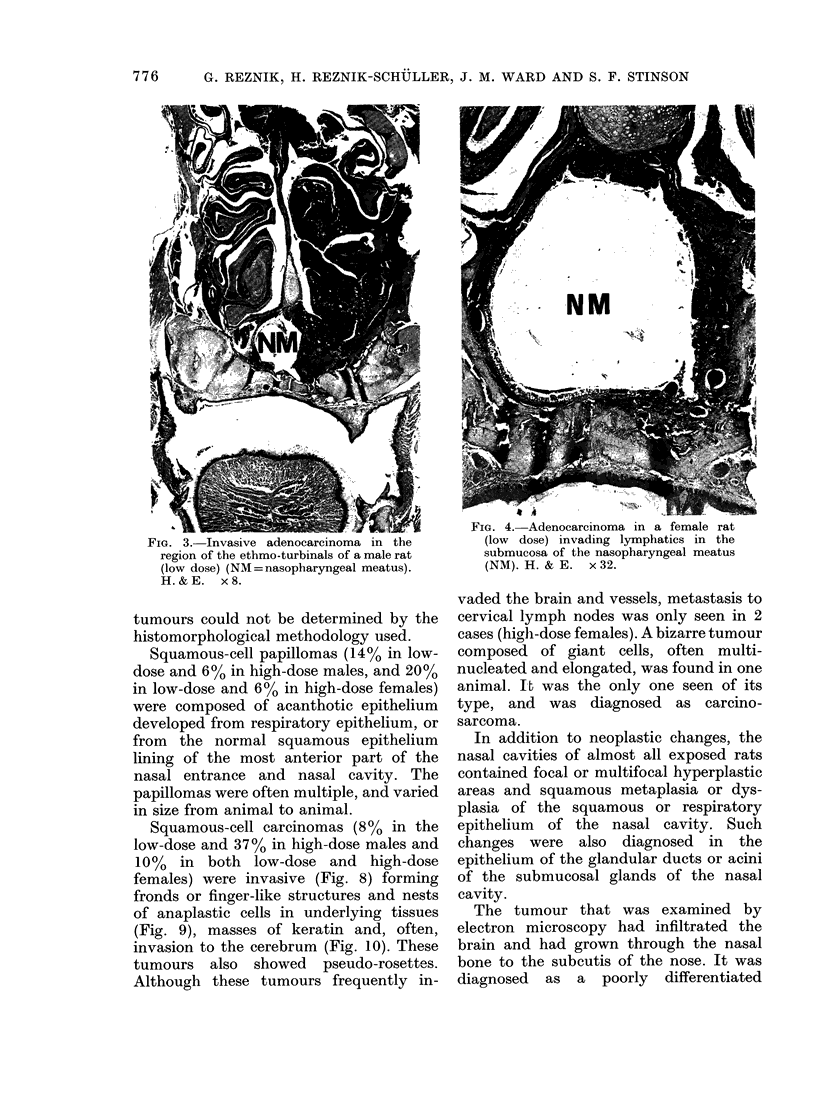

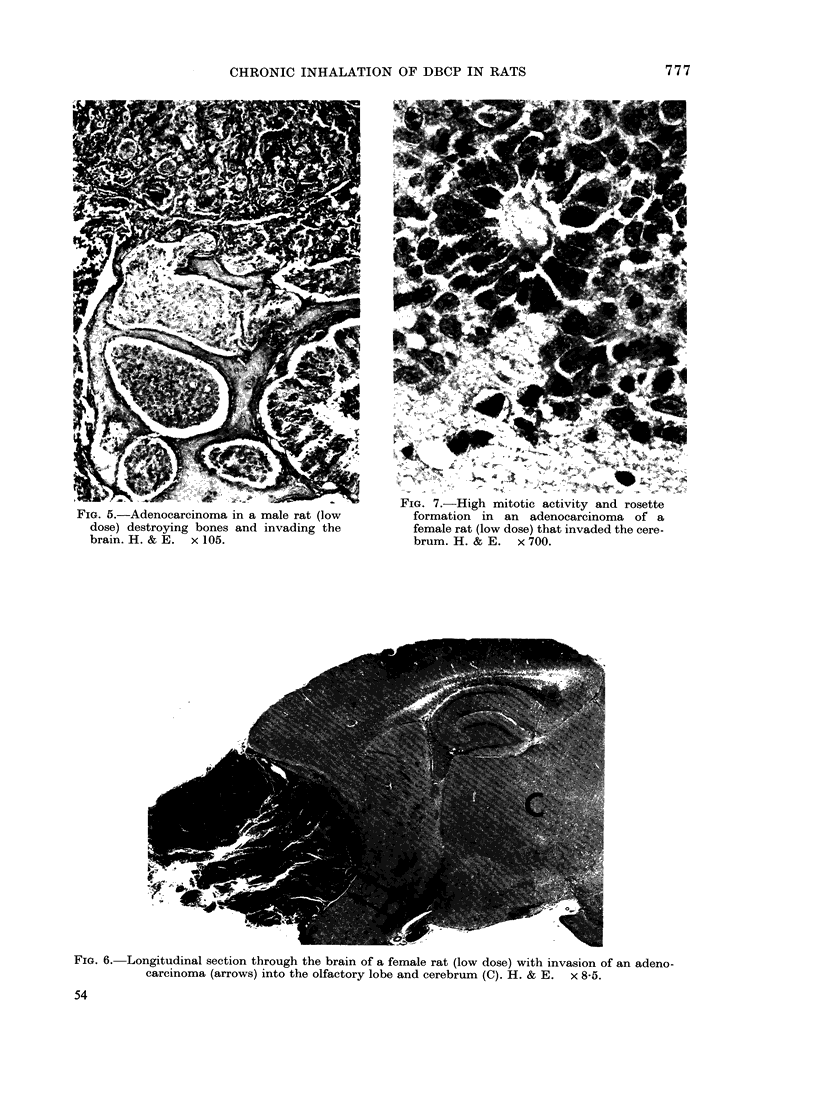

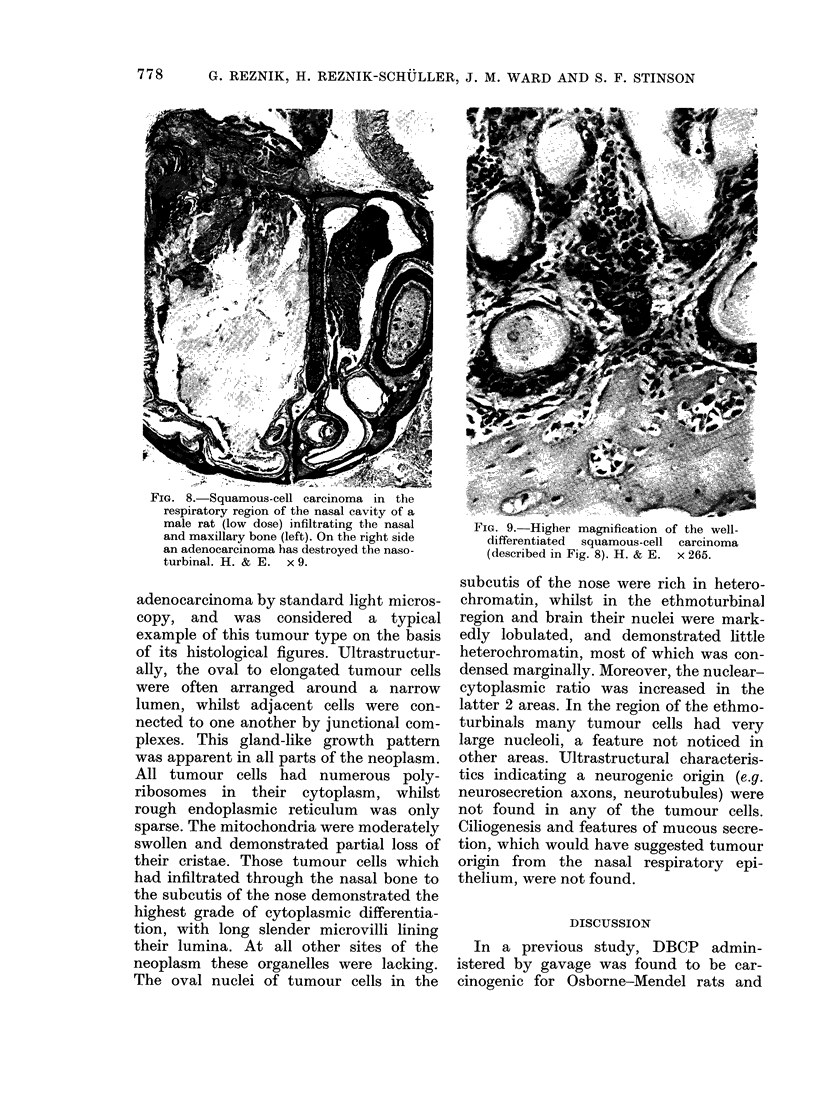

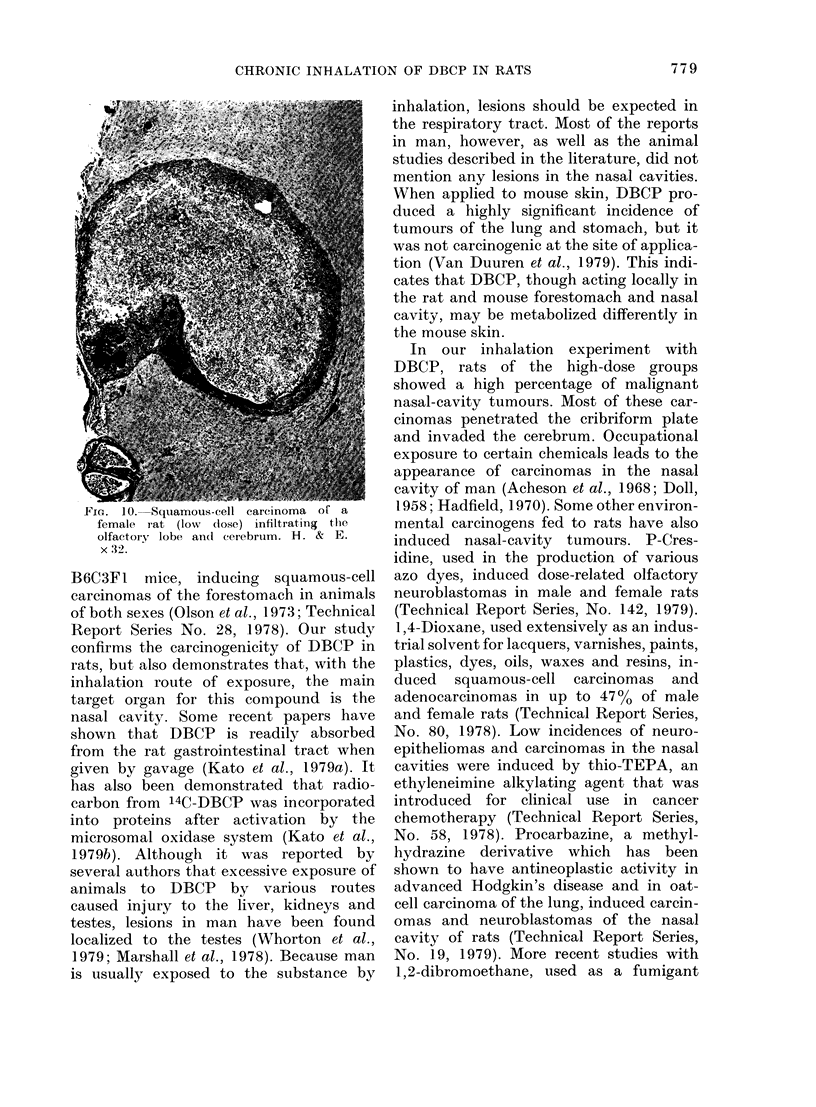

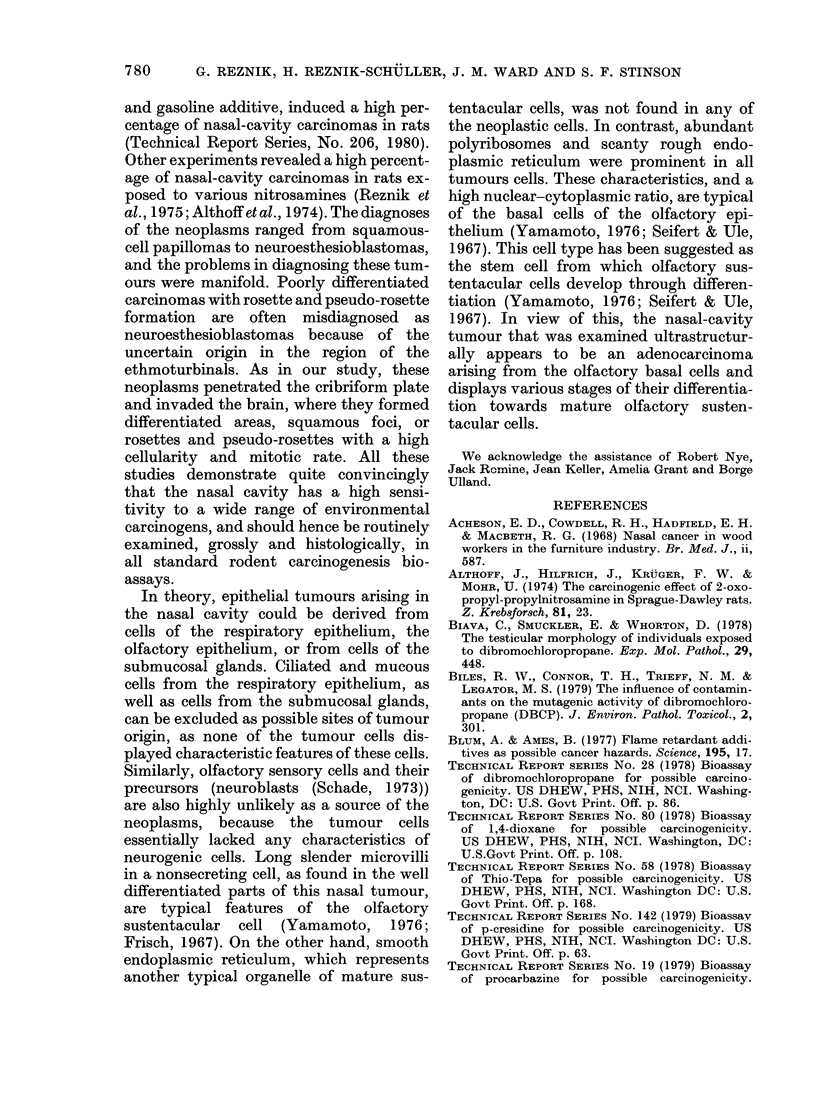

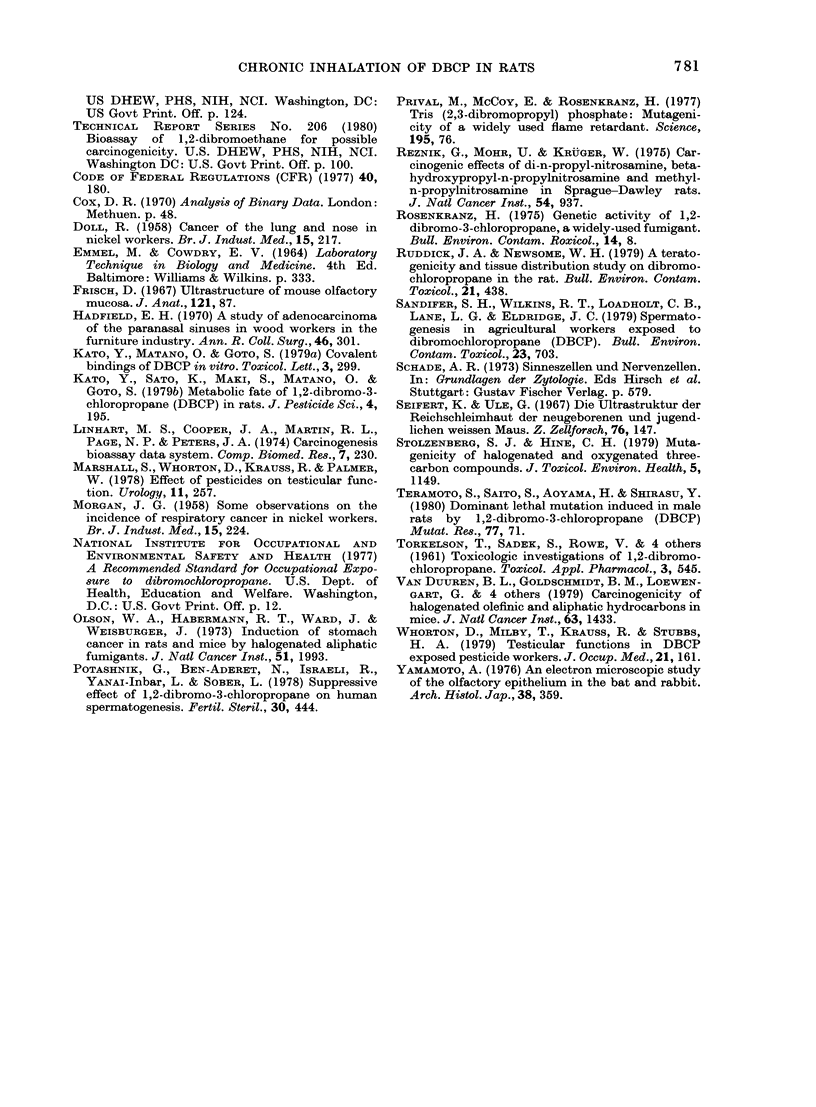


## References

[OCR_00733] Acheson E. D., Cowdell R. H., Hadfield E., Macbeth R. G. (1968). Nasal cancer in woodworkers in the furniture industry.. Br Med J.

[OCR_00745] Biava C. G., Smuckler E. A., Whorton D. (1978). The testicular morphology of individuals exposed to dibromochloropropane.. Exp Mol Pathol.

[OCR_00751] Biles R. W., Connor T. H., Trieff N. M., Legator M. S. (1978). The influence of contaminants on the mutagenic activity of dibromochloropropane (DBCP).. J Environ Pathol Toxicol.

[OCR_00758] Blum A., Ames B. N. (1977). Flame-retardant additives as possible cancer hazards.. Science.

[OCR_00808] DOLL R. (1958). Cancer of the lung and nose in nickel workers.. Br J Ind Med.

[OCR_00817] Frisch D. (1967). Ultrastructure of mouse olfactory mucosa.. Am J Anat.

[OCR_00821] Hadfield E. H. (1970). A study of adenocarcinoma of the paranasal sinuses in woodworkers in the furniture industry.. Ann R Coll Surg Engl.

[OCR_00836] Linhart M. S., Cooper J., Martin R. L., Page N., Peters J. (1974). Carcinogenesis bioassay data system.. Comput Biomed Res.

[OCR_00846] MORGAN J. G. (1958). Some observations on the incidence of respiratory cancer in nickel workers.. Br J Ind Med.

[OCR_00841] Marshall S., Whorton D., Krauss R. M., Palmer W. S. (1978). Effect of pesticides on testicular function.. Urology.

[OCR_00860] Olson W. A., Habermann R. T., Weisburger E. K., Ward J. M., Weisburger J. H. (1973). Induction of stomach cancer in rats and mice by halogenated aliphatic fumigants.. J Natl Cancer Inst.

[OCR_00866] Potashnik G., Ben-Aderet N., Israeli R., Yanai-Inbar I., Sober I. (1978). Suppressive effect of 1,2-dibromo-3-chloropropane on human spermatogenesis.. Fertil Steril.

[OCR_00872] Prival M. J., McCoy E. C., Gutter B., Rosendranz H. S. (1977). Tris(2,3-dibromopropyl) phosphate: mutagenicity of a widely used flame retardant.. Science.

[OCR_00878] Reznik G., Mohr U., Krüger F. W. (1975). Carcinogenic effects of Di-n-propylnitrosamine, beta-hydroxypropyl-n-propylnitrosamine, and methyl-n-propylnitrosamine on Sprague-Dawlay rats.. J Natl Cancer Inst.

[OCR_00885] Rosenkranz H. S. (1975). Genetic activity of 1,2-dibromo-3-chloropropane, a widely-used fumigant.. Bull Environ Contam Toxicol.

[OCR_00896] Sandifer S. H., Wilkins R. T., Loadholt C. B., Lane L. G., Eldridge J. C. (1979). Spermatogenesis in agricultural workers exposed to dibromochloropropane (DBCP).. Bull Environ Contam Toxicol.

[OCR_00913] Stolzenberg S. J., Hine C. H. (1979). Mutagenicity of halogenated and oxygenated three-carbon compounds.. J Toxicol Environ Health.

[OCR_00925] TORKELSON T. R., SADEK S. E., ROWE V. K., KODAMA J. K., ANDERSON H. H., LOQUVAM G. S., HINE C. H. (1961). Toxicologic investigations of 1,2-dibromo-3-chloropropane.. Toxicol Appl Pharmacol.

[OCR_00919] Teramoto S., Saito R., Aoyama H., Shirasu Y. (1980). Dominant lethal mutation induced in male rats by 1,2-dibromo-3-chloropropane (DBCP).. Mutat Res.

[OCR_00929] Van Duuren B. L., Goldschmidt B. M., Loewengart G., Smith A. C., Melchionne S., Seldman I., Roth D. (1979). Carcinogenicity of halogenated olefinic and aliphatic hydrocarbons in mice.. J Natl Cancer Inst.

[OCR_00935] Whorton D., Milby T. H., Krauss R. M., Stubbs H. A. (1979). Testicular function in DBCP exposed pesticide workers.. J Occup Med.

[OCR_00939] Yamamoto M. (1976). An electron microscopic study of the olfactory mucosa in the bat and rabbit.. Arch Histol Jpn.

